# Near-infrared spectroscopy-based cerebral oxygen monitoring in perioperative neurocognitive disorders of older adults: current evidence and future perspectives

**DOI:** 10.3389/fnagi.2026.1809264

**Published:** 2026-04-09

**Authors:** Yuhan Meng, Ruyi Xia, Meiru Jiang, Tao Li, Qiaoxia Sun, Jiahai Ma

**Affiliations:** 1 The Second School of Clinical Medicine, Binzhou Medical University, Yantai, China; 2 School of Anesthesiology, Shandong Second Medical University, Yantai, China; 3 Department of Anesthesiology, Yantai Yuhuangding Hospital, Yantai, China

**Keywords:** cerebral oxygen saturation monitoring, geriatric neurological dysfunction, near-infrared spectroscopy, older adult patients, perioperative neurocognitive disorders

## Abstract

Perioperative neurocognitive disorders (PNDs) are common pathophysiological states and neurological complications in older adult patients after surgery, which severely restrict the quality of postoperative rehabilitation and increase the social medical burden. Cerebral oxygen saturation monitoring using near-infrared spectroscopy (NIRS) has emerged as a valuable tool for perioperative management. Recently, multiregional NIRS monitoring, an extension of this technology, has gained attention for its potential to detect regional cerebral hypoperfusion in multiple vascular territories and provide more comprehensive guidance for anesthesia interventions. This study comprehensively reviews the definition, classification, and clinical characteristics of different subtypes of PND pathogenesis and risk factors, elaborates on the principles and limitations of NIRS monitoring technology, integrates the research progress of NIRS-based multiregional monitoring, analyzes the clinical value of cerebral oxygen saturation monitoring and its application status in different types of surgery, and focuses on exploring the advantages, application prospects, and current core controversies of multiregional cerebral oxygen saturation monitoring technology to provide a reference for the optimization of perioperative anesthesia management and neuroprotective strategies in older adult patients.

## 1 Introduction

With the accelerated process of global population aging, the number of older adult surgical patients is increasing year by year, and perioperative neurocognitive disorders (PNDs) have become an important complication affecting the postoperative prognosis of older adult patients. PND is mainly characterized by memory impairment, decreased abstract thinking ability, disorientation, and reduced social activity ability. In the short term, it can lead to prolonged hospital stays and increased medical expenses; in the long term, it accelerates the frailty process of older adult patients and increases the long-term mortality rate. Although perioperative anesthesia assessment and monitoring technologies have been continuously improved in recent years, which may reduce the incidence of PND, clinical practice still faces multiple challenges, such as unclear pathogenesis, lack of early warning indicators, and limited intervention measures.

## 2 Perioperative neurocognitive disorders

### 2.1 Definition and classification of perioperative neurocognitive disorders

Descriptions of cognitive function changes after general anesthesia can be traced back to more than 100 years ago, which were initially classified into the categories of delirium and dementia. In the 1980s, the concept of “Postoperative cognitive dysfunction (POCD)” was formally proposed to describe the postoperative cognitive decline confirmed by neuropsychological tests. However, this disorder has significant individual differences and heterogeneity in clinical diagnosis. With the further understanding in recent years, it is more accurately and comprehensively generalized as PND (Liang et al., 2025). At present, the internationally recognized classification criteria for perioperative neurocognitive disorders divide it into four categories: preoperatively existing cognitive impairment or delirium, delirium occurring within 7 days after surgery, delayed neurocognitive recovery diagnosed within 30 days after surgery, and cognitive dysfunction occurring from 30 days to 1 year after surgery (Kong et al., 2022). Perioperative neurocognitive disorders are carefully classified according to different time points to accurately describe the changes in patients’ cognitive function at each stage. Since this study focuses on postoperative cognitive function changes, postoperative delirium, delayed neurocognitive recovery, and classic POCD are collectively classified into the category of PND for discussion. With the intensification of population aging, PND has currently become a public health issue of global concern, posing a major challenge to the anesthesia of older adult surgical patients (Khachaturian et al., 2020) (see Table 1).

**Table 1:** Clinical characteristics of different subtypes of perioperative neurocognitive disorders (PNDs) in older adult surgical patients.

PND subtype	Diagnostic criteria	Onset time	Main confounding factors	Assessment tools
Postoperative delirium	DSM-5/ICD-11 diagnostic criteria, acute onset of attention and consciousness disturbance, fluctuating course	Within 7 days after surgery	Intraoperative hypotension, cerebral hypoperfusion, postoperative pain, and sleep disturbance	CAM, DRS-R-98, and DCT
Delayed neurocognitive recovery	Persistent cognitive decline after the acute phase, no brain structural damage, and reversible in the short term	Within 30 days after surgery	Neuroinflammation, mitochondrial dysfunction, and neurotransmitter imbalance	MoCA, MMSE, and RBANS
Long-term cognitive decline	Persistent cognitive impairment for more than 3 months, potentially irreversible and progressive	30 days to 1 year after surgery (even longer)	β-amyloid deposition, excessive phosphorylation of tau protein, and chronic neuroinflammation	MoCA, MMSE, and neuroimaging [magnetic resonance imaging (MRI)/positron emission tomography (PET)]

DSM-5, Diagnostic and Statistical Manual of Mental Disorders, 5th Edition; ICD-11, International Classification of Diseases, 11th Revision; CAM, Confusion Assessment Method; DRS-R-98, Delirium Rating Scale-Revised-98; DCT, Delirium Cognitive Test; MoCA, Montreal Cognitive Assessment; MMSE, Mini-Mental State Examination; RBANS, Repeatable Battery for the Assessment of Neuropsychological Status.

### 2.2 Pathogenesis and risk factors of perioperative neurocognitive disorders

The incidence and onset time of PND show a fluctuating process in the long run. Generally, it gradually decreases from several weeks after surgery to 3 months and 12 months after surgery. However, according to the results of long-term follow-up by some scholars, the incidence will rise again 5 years after surgery and even evolve into long-term neurocognitive decline, increasing the postoperative mortality rate (Atkins et al., 2024; Li et al., 2023). The pathogenesis of PND is complex and has not been fully clarified yet. Existing studies have shown that its occurrence may be related to the following core mechanisms: ① Surgical trauma and anesthetic drugs can activate microglia in the central nervous system, promote the release of inflammatory factors such as IL-1β, and trigger inflammatory cascade reactions and oxidative stress reactions. At the same time, excessive production of reactive oxygen species (ROS) damages the redox homeostasis of neurons, leading to the formation of a neuroinflammatory microenvironment (Yang et al., 2024). ② Mitochondria play a key role in maintaining the energy metabolism and redox balance of neurons. Studies have shown that perioperative stress can cause abnormal mitochondrial structure and function, which, in turn, induce oxidative stress, decreased ATP production, and neuronal damage. These changes are closely related to PND. Mitochondrial dysfunction is considered an early key pathological mechanism of various cognitive disorders, including PND (Liu et al., 2025; You et al., 2024). ③ The occurrence of PND is also closely related to the changes in synaptic structure and function. For example, abnormalities in the brain-derived neurotrophic factor (BDNF)/tropomyosin-related kinase (TrkB) signaling pathway can affect the function of the hippocampal-medial prefrontal cortex (HPC-mPFC) pathway, which plays a key role in learning and memory; abnormal downstream signals mediated by glutamate receptors are also associated with cognitive impairment (Ge et al., 2023). In addition, the imbalance of neurotransmitter systems, such as acetylcholine, glutamate, and gamma-aminobutyric acid (GABA), has also been reported to be involved in the pathological process of PND (Zhang Z. et al., 2024). ④ Neurodegenerative changes such as β-amyloid deposition and hyperphosphorylated tau protein aggregation play an important role in the development of PND, suggesting that they may become potential therapeutic targets. Perioperative complications, long-term mortality, infection, and other factors provide support for the above studies, but these factors still cannot accurately describe the pathophysiological characteristics of PND, and further research is needed (Evered et al., 2022). Risk factors of PND need to be identified throughout the perioperative process: preoperative factors include age (≥65 years old), low educational level, hypertension, diabetes, decreased cognitive reserve, and other underlying diseases; intraoperative factors include surgical type (cardiac surgery and large vascular surgery have higher risks), intraoperative hypotension, cerebral hypoperfusion, impaired cerebrovascular autoregulation function, selection of anesthetic drugs, and management of anesthesia depth (Yang et al., 2022). A meta-analysis by Miravalles et al. (2025) confirmed that intraoperative application of cholinergic receptor blockers, such as scopolamine, can induce memory impairment and significantly increase the risk of PND. In recent years, mechanistic studies have shown that circadian rhythm disturbance leads to PND through two interrelated pathways: neuroinflammation and glymphatic system dysfunction. Correcting postoperative circadian rhythm disturbance has gradually become a new research direction to improve the occurrence of PND. Therefore, a comprehensive screening process for high-risk groups of PND should be integrated into every link of clinical diagnosis and treatment to provide a reference for formulating surgical and anesthetic plans (Campbell and Figueiro, 2024).

### 2.3 Anesthesia management measures for perioperative neurocognitive disorders

**Figure 1:**
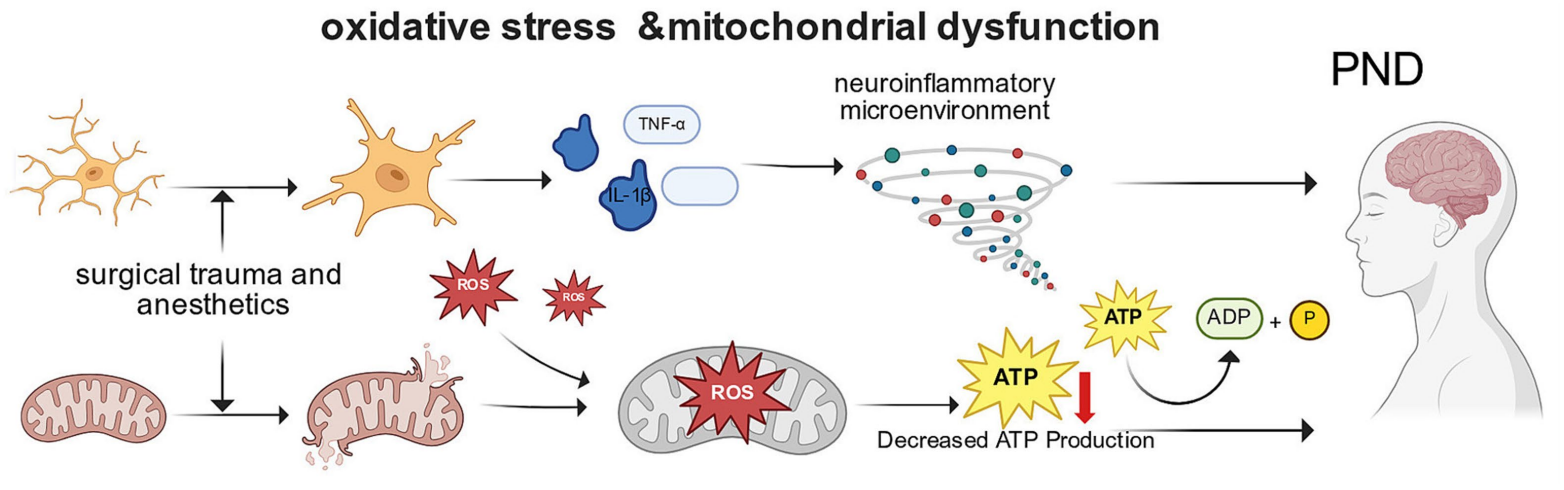
Schematic diagram of oxidative stress and mitochondrial dysfunction in the pathogenesis of PND in older adult surgical patients. Core information: Perioperative stress (surgical trauma/anesthetic drugs) induces abnormal mitochondrial structure and function, leading to excessive production of ROS and neuroinflammatory response, which is a key early mechanism for the occurrence of PND in older adult patients; targeting mitochondrial homeostasis may become a potential therapeutic strategy for PND (Yang et al., 2024; Liu et al., 2025; You et al., 2024). ROS, reactive oxygen species; ATP, adenosine triphosphate; IL-1β, interleukin-1β; TNF-α, tumor necrosis factor-α.

**Figure 2:**
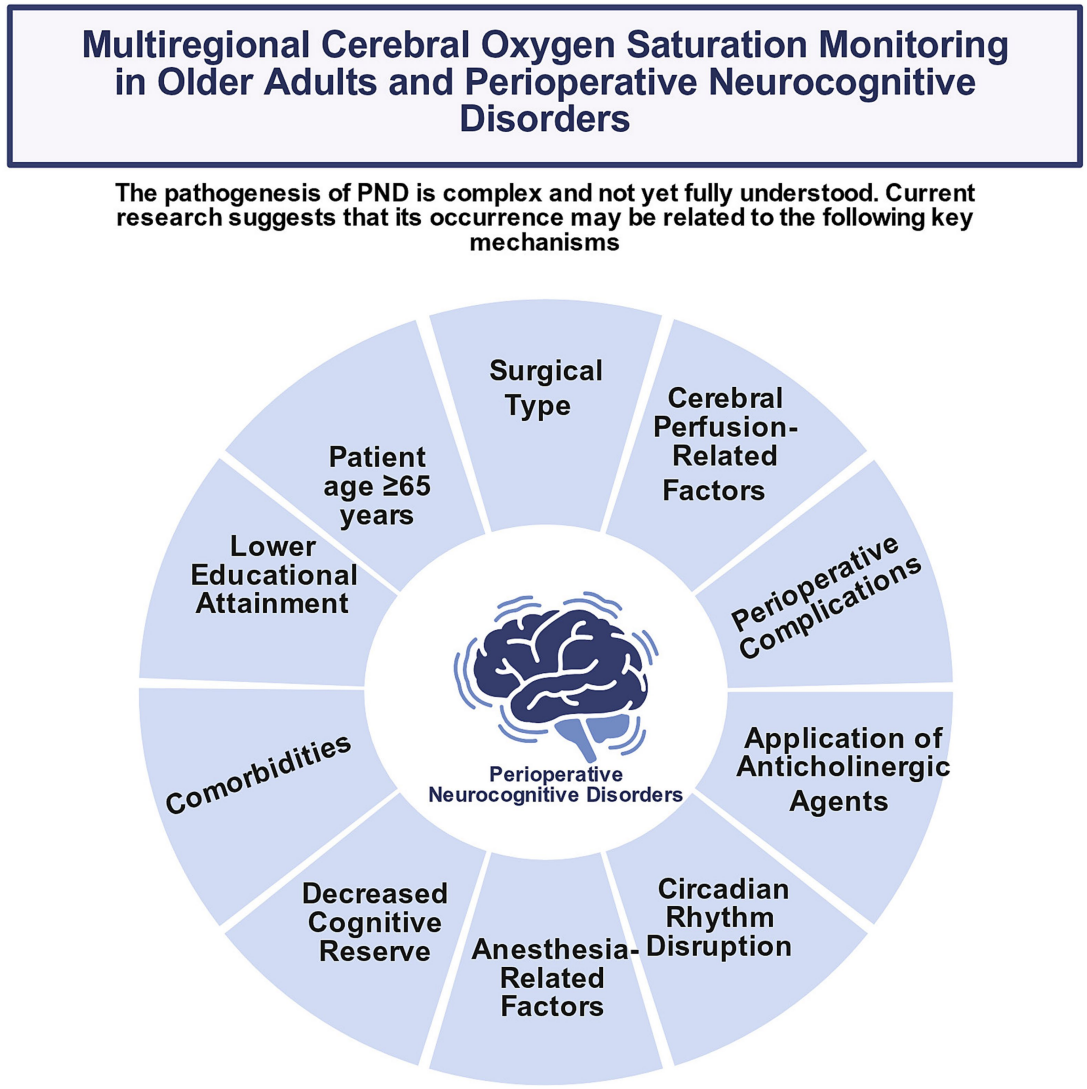
Schematic diagram of risk factors for perioperative cognitive dysfunction in older adult surgical patients. Core information: PND is a complication induced by multiple factors. Preoperative age and underlying diseases, intraoperative cerebral hypoperfusion and high-risk surgical types, and postoperative neuroinflammation and sleep disturbance are the core risk factors; comprehensive intervention of multi-stage risk factors may reduce the incidence of PND. References: Preoperative factors (Yang et al., 2022); intraoperative factors (Yang et al., 2022; Han et al., 2025; Zhu et al., 2021); postoperative factors (Campbell and Figueiro, 2024; Cui et al., 2021).

The existing whole-process strategies for the prevention and treatment of postoperative neurological dysfunction in older adult patients include comprehensive geriatric assessment, anesthesia monitoring and management based on the concept of Enhanced Recovery After Surgery (ERAS) during surgery, and multimodal analgesia after surgery (Kotekar et al., 2018). A variety of existing preoperative assessment tools for PND include the Mini-Mental State Examination, Montreal Cognitive Assessment, Repeatable Battery for the Assessment of Neuropsychological Status, Digit Span Test, Confusion Assessment Method, Delirium Rating Scale-Revised-98, and Delirium Cognitive Test. The above assessment methods are helpful for indicating the risk of PND, but such scales lack the support of objective biological indicators and need to be comprehensively judged in combination with patients’ clinical characteristics (Anand et al., 2024). The selection of anesthesia methods and the application of anesthetic drugs also affect the occurrence of PND to a certain extent. A study by Wang et al. (2022) (2022, randomized controlled trial (RCT), sample size *n* = 96, older adult patients undergoing abdominal surgery) found that the use of dexmedetomidine during the induction and maintenance of clinical anesthesia can reduce the incidence of early postoperative delirium (assessed within 3 days) in older adult patients and alleviate perioperative stress and inflammatory reactions. In recent years, based on the principle of maintaining the balance of cerebral oxygen supply and demand during surgery and reducing the occurrence of cerebral hypoxia events, as well as the development of NIRS technology, monitoring of cerebral oxygen saturation has been gradually applied in perioperative anesthesia management, which may potentially reduce the incidence of PND. However, the association and correlation between cerebral oxygen saturation monitoring and PND have not been clarified, and further clinical trials are needed (Holmgaard et al., 2019). Although the occurrence and development of PND are self-limiting, the current clinical treatment methods are limited, and the importance of prevention is lacking. Therefore, the future development direction should focus on looking for relevant biomarkers; exploring emerging predictive factors and therapeutic targets to support early diagnosis, prognostic assessment, and risk stratification; developing effective drugs; and monitoring management measures to potentially reduce the incidence of PND and improve the postoperative quality of life of patients (see Figures 1, 2).

## 3 Multiregional cerebral oxygen saturation monitoring based on near-infrared spectroscopy technology

### 3.1 Near-infrared spectroscopy technology

Near-infrared spectroscopy (NIRS) has gradually been applied in perioperative cerebral oxygen saturation detection by virtue of its advantages of non-invasiveness, real-time, and continuous monitoring, providing a new technical path for maintaining the balance of cerebral oxygen supply and demand. However, traditional NIRS monitoring is mostly limited to the frontal lobe region, which makes it difficult to cover other brain regions such as the temporal lobe and parietal lobe, easy to miss local cerebral hypoperfusion or oxygenation abnormalities, and affects the sensitivity and specificity of monitoring. As an important extension of NIRS technology, multiregional cerebral oxygen saturation monitoring realizes the synchronous monitoring of oxygenation status in multiple brain regions through a multi-channel design, breaking through the limitations of traditional single-region monitoring. Based on existing research evidence, this study comprehensively sorts out the pathophysiological characteristics of PND and the development course of NIRS monitoring technology and focuses on analyzing the application value and research progress of multiregional cerebral oxygen saturation monitoring in the prevention of PND in older adult patients to provide a theoretical basis for clinical practice and subsequent research. The physical basis of NIRS lies in the fact that near-infrared light, usually with a wavelength range of 700–900 nm, can penetrate biological tissues, and oxygenated hemoglobin (HbO₂) and deoxygenated hemoglobin (HHb) have different absorption characteristics in this band, thus allowing the calculation of tissue oxygenation status through light attenuation (Sherimon et al., 2025). In the current medical field, maintaining sufficient tissue oxygen supply is the premise of oxygen metabolism, and the core goal of treatment is to maintain, restore, or optimize tissue oxygenation. However, in routine clinical practice, evaluating tissue oxygenation to detect occult local ischemia is still a challenge (Scheeren et al., 2012). With the development of science and technology, NIRS technology has been applied in an increasingly wide range of scenarios and surgical types. Its principle is that near-infrared light can penetrate biological tissues and obtain real-time, non-invasive information related to tissue oxygenation and metabolism. At present, its application in monitoring regional cerebral oxygen saturation (rScO₂) in cardiothoracic surgery and carotid endarterectomy has achieved a certain value in predicting postoperative neurocognitive prognosis. A study on older adult patients undergoing cardiac surgery (Ali et al., 2022; Shaaban-Ali et al., 2021) compared the average memory scores of the cerebral oxygen monitoring intervention group and the non-intervention group, and the results showed that the average memory score of the intervention group was higher at 6 months after surgery, which means that the postoperative neurocognitive function recovery was better than that of the non-cerebral oxygen monitoring group. NIRS technology uses the different absorption spectra of oxygenated hemoglobin and deoxygenated hemoglobin in the infrared wavelength range to measure the contents of oxygenated hemoglobin and deoxygenated hemoglobin and analyze the ratio of tissue oxygen absorption to oxygen delivery to reflect the oxygen uptake of the monitored tissue (Marin and Moore, 2024). NIRS is a reliable method for monitoring cerebral oxygen saturation, and it has significant advantages in stability and reliability compared with traditional pulse oximeters under conditions such as low temperature and hypoxia (Alevizakos et al., 2024).

### 3.2 Limitations of near-infrared spectroscopy technology

However, NIRS technology also has certain limitations. Its monitoring is easily interfered with by skin blood flow and temperature, adipose tissue thickness, jaundice, and myoglobin. At the same time, conventional NIRS monitoring can only cover the frontal lobe region and cannot cover the lateral brain, the posterior brain, and other parts, which may miss ischemia or oxygenation abnormalities in these regions; in addition, the monitoring area under the sensor is small, making it difficult to reflect the overall tissue oxygenation status, which needs to be improved by relying on multi-channel NIRS. Therefore, this study will focus on analyzing the relevant studies of multi-brain region and multi-channel NIRS monitoring in recent years to explore the effect of multi-brain region cerebral oxygen monitoring in preventing the occurrence of postoperative cognitive dysfunction.

### 3.3 Important clinical value of cerebral oxygen saturation monitoring

Maintaining normal brain function is inseparable from sufficient oxygen supply. As a high-metabolism organ of the human body, the brain accounts for more than 20% of the total body oxygen consumption. At the same time, the brain has a variety of homeostatic mechanisms to maintain a constant blood supply to meet its high metabolic demand. Cerebral blood flow-pressure autoregulation is such a mechanism. Through this mechanism, cerebral blood flow remains stable within a certain blood pressure range to ensure the continuous supply of oxygenated blood. When the blood pressure is lower or higher than the autoregulation range, cerebral blood flow is pressure-dependent, which can easily lead to cerebral ischemia or cerebral congestion, respectively (Hogue et al., 2021). Paying attention to cerebral oxygenation is an important research content of clinical anesthesia in recent years. The latest international consensus on the application of perioperative cerebral oxygen monitoring points out that cerebral oxygen saturation monitoring can be used to identify high-risk groups of adverse outcomes in patients undergoing cardiac surgery (Fischer-Kumbruch et al., 2022). At present, there are various methods for monitoring cerebral oxygen saturation, each with its own advantages and disadvantages, mainly including NIRS monitoring, jugular venous bulb oxygen saturation monitoring, brain tissue oxygen partial pressure monitoring, and other methods. In recent years, emerging monitoring methods have also been developed, such as electroencephalography, positron emission tomography, functional magnetic resonance imaging, and transcranial Doppler ultrasound (Zhang C. Y. et al., 2024). Among them, NIRS is an emerging non-invasive cerebral oxygenation monitoring technology that can conduct continuous and real-time monitoring of cerebral oxygen saturation, helping anesthesiologists to early identify the imbalance of cerebral oxygen supply and demand and cerebral hypoxia. According to a recent meta-analysis (Qiu et al., 2025) (2025, meta-analysis of randomized controlled trials, sample size *n* = 1,876, older adult patients undergoing non-cardiac surgery), it is shown that the application of optimized anesthesia management based on intraoperative cerebral oxygen saturation monitoring in older adult patients undergoing elective non-cardiac surgery under general anesthesia can significantly reduce the incidence of PND within 7 days after surgery and may reduce the occurrence of PND at 3 months and even longer after surgery. However, in cardiac surgery, Semrau et al. (2021) (2021, review, sample size *n* = 3,217, older adult patients undergoing cardiac surgery) concluded through research that only 8 (47%) of the 17 included observational studies found a significant correlation between the occurrence of rScO₂ cerebral oxygen saturation decrease events measured by NIRS during cardiac surgery and postoperative neurological complications; only 3 (30%) of the 10 interventional studies supported that the use of NIRS during cardiac surgery can reduce the incidence of postoperative injury. In summary, observational data mostly support the association between cerebral oxygen saturation and PND, but randomized controlled trials have not fully confirmed the direct causal relationship between decreased cerebral oxygenation and delirium, PND, or stroke, suggesting that preoperative low cerebral oxygen saturation may be a surrogate indicator of cognitive vulnerability or poor cerebral reserve function.

It is generally believed that cerebral oxygen desaturation may be another risk factor for adverse neurological outcomes, so monitoring perioperative cerebral oxygen saturation is of great significance. In terms of monitoring thresholds, the normal range of cerebral oxygen saturation is 58–82%. Clinical data show that a decrease in rScO₂ by more than 20% compared with the baseline may lead to cerebral oxygen desaturation events and the occurrence of PND due to severe cerebral hypoperfusion. At the same time, a perioperative absolute rScO₂ value lower than 50–60% is associated with increased adverse neurological outcomes and mortality (Tsaousi et al., 2021). Cerebral oxygen saturation monitoring is also currently applied to monitor cerebral oxygen during cardiopulmonary resuscitation (CPR). By monitoring the oxygen delivery and uptake of the brain during CPR, cerebral oximeters can real-time provide information on the ischemic load and oxygenation of important organs, providing opportunities to improve the quality of resuscitation and prevent ischemia/reperfusion injury. Research data show that rScO₂ ≥40% can increase the probability of return of spontaneous circulation, rScO₂ ≥50% may produce a neuroprotective effect, and the neuroprotective effect is more significant when the proportion of time maintaining this level during CPR is ≥60%. This “40-50-60” principle is used as a multimodal strategy for cerebral resuscitation in clinical application (Huppert and Parnia, 2022).

### 3.4 Application status of cerebral oxygen saturation monitoring

Based on the advantages of non-invasiveness and real-time monitoring of NIRS technology, cerebral oxygen saturation monitoring has been widely explored and applied in the perioperative period of multidisciplinary surgery. Due to the differences in hemodynamic characteristics, influencing factors of cerebral perfusion, and patient groups in different surgical types, the clinical value and application scenarios of cerebral oxygen monitoring show significant specificity. Combined with recent clinical research evidence, the application status and practical value are comprehensively sorted out from multiple surgical fields as follows.

#### 3.4.1 Application in cardiac surgery

Cardiac surgery is a common factor for the occurrence of PND in older adult patients. More than 50% of patients undergoing cardiac surgery can be detected with cognitive decline at discharge, and the core inducement is the occurrence of ischemic events caused by cerebral hypoperfusion during cardiac surgery (Moore et al., 2022). Current perioperative cerebral oxygen monitoring studies mainly focus on patients undergoing cardiac surgery, but the conclusions are somewhat different. According to a study by Han et al. (2025) (2025, randomized controlled trial, single center, sample size *n* = 208, older adult patients undergoing off-pump coronary artery bypass grafting), it was found that there was no significant difference in the incidence of complications within 30 days after surgery, including brain, heart, respiratory, kidney, infection-related complications, and death, between the NIRS monitoring cerebral oxygen-guided postoperative care group and the conventional care group in patients undergoing off-pump coronary artery bypass grafting, but the incidence of postoperative delirium in the NIRS group was lower (12.5% vs. 21.6%), suggesting that this technology may have potential value in predicting specific neurological-related outcomes such as PND. In a study by Heuer et al. (2025) on cerebral oxygen monitoring for predicting the occurrence of stroke after acute type A aortic dissection surgery (2025, cohort study, sample size *n* = 89, older adult patients with acute type A aortic dissection), it was found that decreased cerebral oxygen saturation during deep hypothermic cardiopulmonary bypass is a key risk factor for stroke, and rScO₂ <50% can be used as a critical early warning threshold, suggesting the need to strengthen cerebral perfusion or deepen hypothermic protection. The reason for the different conclusions of the above two studies may be the influence of cardiopulmonary bypass application in cardiac surgery, but in general, the application of NIRS technology for monitoring in cardiac surgery is of great significance.

Due to the risk and complexity of the perioperative period, older adult patients undergoing cardiac surgery still experience a persistent decrease in cerebral oxygen saturation after surgery. According to a study by Cioccari et al. (2021) (2021, prospective observational study, sample size *n* = 64, older adult patients undergoing cardiac surgery), the baseline value of cerebral oxygen saturation in the cardiac surgery group decreased from 63.7 to 61.0% after the patients were transferred to the intensive care unit after surgery, and the cerebral oxygen saturation continued to decrease from the second to the seventh day after surgery, with a cerebral oxygen saturation of 53.5% at discharge, which was significantly lower than the preoperative baseline level. However, there was no significant difference in postoperative rScO₂ compared with the baseline in the non-cardiac surgery control group. The potential mechanism and recovery time of such cerebral oxygen saturation decrease need further study, and it also suggests the guiding significance of cerebral oxygen monitoring in the treatment and recovery stage after surgery.

#### 3.4.2 Application in thoracic surgery

With the development of minimally invasive thoracoscopic technology, lung isolation technology is widely used in anesthesia for thoracic surgery, but prolonged one-lung ventilation may cause lung injury and brain injury, significantly increasing the postoperative risk of older adult patients (Teng et al., 2024). The application value of NIRS-based cerebral oxygen monitoring in thoracic surgery has gradually attracted attention. A study by Roberts et al. (2021) (2021, prospective cohort study, sample size *n* = 112, older adult patients undergoing thoracic surgery with one-lung ventilation) found that perioperative management guided by rScO₂ combined with lung protective ventilation can effectively reduce postoperative delirium and shorten hospital stay, but there was no significant advantage in the recovery of cognitive function in the short term after surgery (within 3 days), and intraoperative cerebral oxygen saturation decrease can be used as a high-risk factor for the occurrence of intraoperative and cardiovascular and cerebrovascular adverse events. At present, there are few studies on the cerebral oxygen monitoring threshold in thoracic surgery. According to a study by Cui et al. (2021) (2021, prospective cohort study, sample size *n* = 95, older adult patients undergoing thoracotomy with one-lung ventilation), 20% of patients developed delirium after one-lung ventilation, and a left cerebral oxygen saturation lower than 90% of the baseline or a right cerebral oxygen saturation lower than 85% of the baseline for more than 15 s may be associated with an increased risk of postoperative delirium. Current studies on cerebral oxygen monitoring in thoracic surgery mostly focus on the prediction of short-term postoperative delirium, and there is still a lack of research on the correlation between changes in cerebral oxygen saturation and long-term postoperative cognitive function recovery, so large-scale long-term follow-up studies need to be further carried out.

#### 3.4.3 Application in neurocritical care and neurosurgical surgery

The application of near-infrared spectroscopy (NIRS) cerebral oxygen monitoring in neurocritical care and neurosurgical surgery has been gradually expanded. Its characteristics of non-invasive, continuous, and real-time monitoring of brain tissue oxygen saturation provide important references for clinical decision-making. In brain arteriovenous malformation resection, brain tumor resection, and neurointerventional therapy, such as endovascular therapy for acute ischemic stroke, it can real-time reflect the cerebral oxygenation status; the ratio of brain tissue oxygen partial pressure to arterial oxygen partial pressure is more reliable than the absolute value in identifying hypoxia (Badenes et al., 2016). In anterior surgery for cervical spondylotic myelopathy, the combination of NIRS and somatosensory/motor evoked potentials constitutes multimodal neurophysiological monitoring, which is helpful for the early discovery of spinal cord injury and intervention (Rebelo et al., 2024). In patients with traumatic brain injury, NIRS can identify hypoxia, hypoxemia, or cerebral ischemic events caused by cerebral blood flow disorders and assist in the early diagnosis of intracranial hemorrhage or cerebrovascular autoregulation disorders. NIRS shows the unique advantage of non-invasive continuous monitoring in neurocritical care and neurosurgical surgery, especially having auxiliary value in scenarios such as CEA shunt decision-making, individualized blood pressure management in neurocritical illness, and assessment of consciousness disorders in older adults. However, its application needs to be strictly combined with clinical background, multimodal monitoring data, and a standardized interpretation process to avoid isolated reliance on values (Rivera-Lara et al., 2019).

#### 3.4.4 Application in other surgeries

With the popularization of NIRS technology, cerebral oxygen saturation monitoring has been gradually applied to various surgical types such as orthopedics, carotid endarterectomy, and abdominal surgery. In orthopedic surgery, a study by Zhu et al. (2021) (2021, randomized controlled trial, sample size *n* = 80, older adult patients undergoing knee/hip arthroplasty and spinal surgery) found that in surgeries such as joint surgery (total knee arthroplasty and total hip arthroplasty) and spinal surgery, cerebral oxygen saturation monitoring was significantly associated with cognitive function at 3 months after surgery, and the incidence of delayed neurocognitive recovery in the NIRS-guided group was lower (15% vs. 30%). In carotid endarterectomy, cerebral oxygen monitoring has gradually replaced the traditional transcranial Doppler ultrasound or somatosensory evoked potential methods due to its simple interpretation and high reliability in judging shunt tube placement. At the same time, when the influencing factors such as arterial oxygen saturation and hemoglobin are stable, the fluctuation of rScO₂ can reflect the change of cerebral blood flow, and continuous monitoring of rScO₂ changes can timely find and prevent cerebral ischemia (Sihotsky et al., 2020; Nuermaimaiti et al., 2025). In abdominal surgery, according to a study by Song et al. (2022) (2022, prospective study, sample size *n* = 78, older adult patients undergoing major abdominal surgery), it was found that the rScO₂ measured by the hypercapnia test at the end of surgery and the change trend of postoperative rScO₂ in older adult patients undergoing major abdominal surgery were associated with postoperative delirium, and these indicators can be used as a practical method to predict postoperative delirium, helping to identify patients in the need of early psychological intervention and management (see Table 2).

**Table 2:** Summary of key clinical studies on cerebral oxygen saturation monitoring in different surgical types.

Surgical type	Author and year	Study design	Sample size	Main conclusions
Off-pump coronary artery bypass grafting	Han et al. (2025)	RCT	208	The incidence of postoperative delirium in the NIRS group was lower (12.5% vs. 21.6%), with no significant difference in overall complications
Acute type A aortic dissection surgery	Heuer et al. (2025)	Cohort study	89	rScO₂ <50% during deep hypothermic cardiopulmonary bypass is a key risk factor for postoperative stroke
Thoracic surgery with one-lung ventilation	Roberts et al. (2021)	Cohort study	112	rScO₂-guided management can reduce postoperative delirium and shorten hospital stay
Geriatric orthopedic surgery	Zhu et al. (2021)	RCT	80	The incidence of delayed neurocognitive recovery in the NIRS-guided group was lower (15% vs. 30%)
Geriatric major abdominal surgery	Song et al. (2022)	Prospective study	78	rScO₂ from the hypercapnia test at the end of surgery can predict postoperative delirium

### 3.5 Multiregional cerebral oxygen saturation monitoring

**Figure 3:**
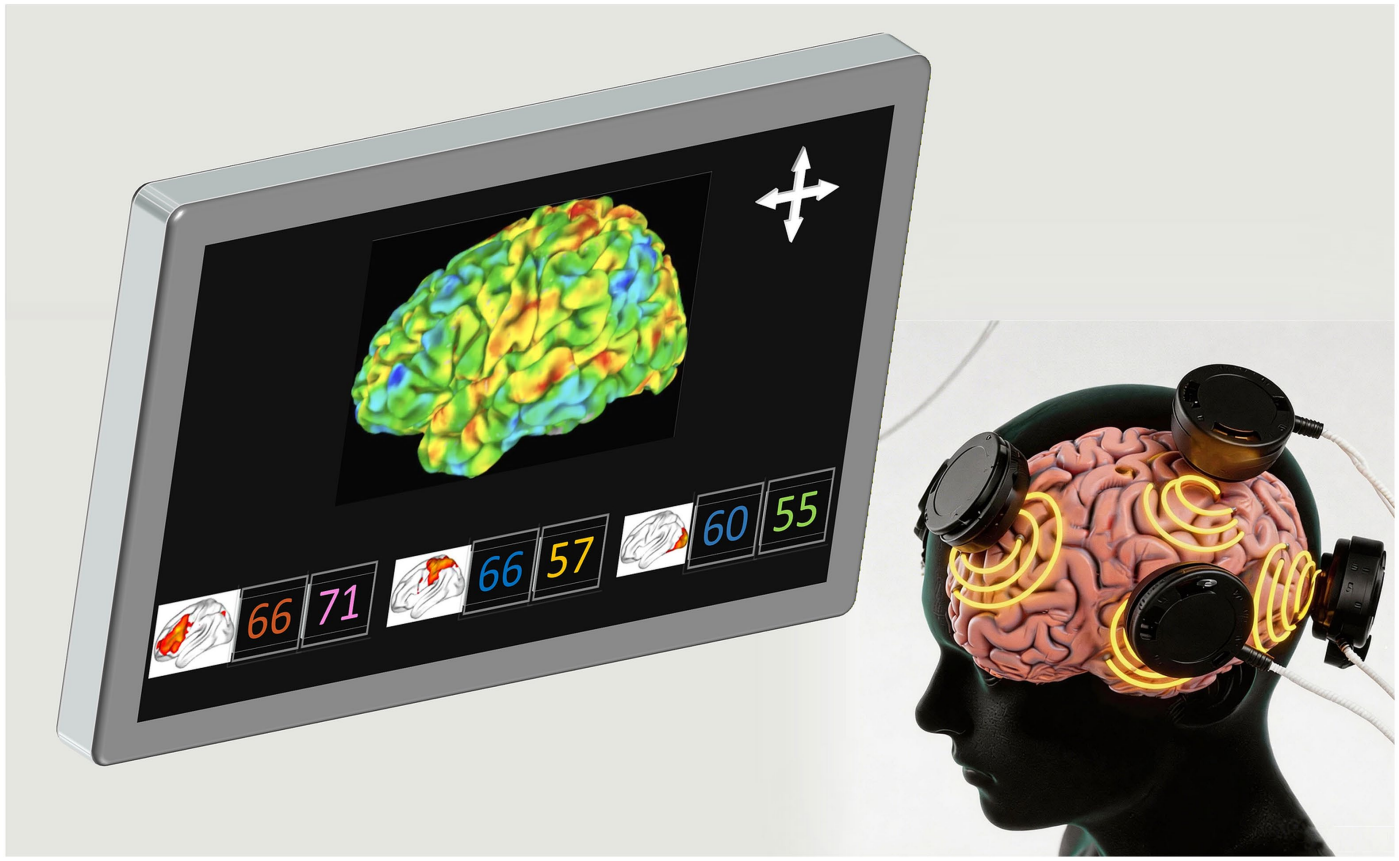
Schematic diagram of NIRS-based multiregional cerebral oxygen monitoring in older adult surgical patients. Monitoring regions: prefrontal lobe, parietal lobe, temporal lobe, and occipital lobe. Monitoring principle: multi-channel NIRS sensors are placed in different brain regions to collect real-time rScO_2_ data, and the central system integrates and analyzes the oxygenation status of each region. Core information: Multiregional cerebral oxygen monitoring realizes oxygenation monitoring of the whole brain region by expanding the sensor coverage, which can avoid the missed detection of hypoxia in the non-prefrontal brain regions and improve the sensitivity and specificity of cerebral oxygen monitoring (Kamar et al., 2024; Rummel et al., 2018). NIRS, near-infrared spectroscopy; rScO_2_, regional cerebral oxygen saturation.

Cerebral oximeters have been widely used to evaluate brain tissue oxygen saturation and monitor the occurrence of cerebral hypoxia events during surgery. However, currently commonly used local cerebral oximeters usually only have two channels for monitoring cerebral oxygen saturation in the bilateral frontal lobes (Kamar et al., 2024). The blood composition in this area is approximately 75% venous blood, 20% arterial blood, and 5% capillary blood, mainly from the watershed area of the anterior cerebral artery and the middle cerebral artery. Thus, the obtained brain tissue oxygenated hemoglobin saturation is mainly derived from the cerebral cortical gray matter, reflecting the balance of oxygen supply and oxygen consumption in this area. This limited spatial coverage may lead to undetected cerebral oxygen desaturation events in other parts. Due to the differences in vascular blood supply characteristics and metabolic demands of different brain regions, single-region monitoring is easy to miss local oxygenation abnormalities in parts such as the temporal lobe and parietal lobe, leading to false-negative results (Scheeren et al., 2012). The core advantage of multiregional cerebral oxygen monitoring is to break through the limitations of traditional single-brain region monitoring. It can conduct continuous and real-time monitoring of cerebral oxygen saturation in multiple anatomical regions of the brain, such as the left and right frontal lobes, parietal lobes, temporal lobes, and occipital lobes, comprehensively reflect the oxygen supply and oxygen consumption balance status of different regions of the whole brain, and provide a more accurate and comprehensive brain function assessment basis for clinical diagnosis and treatment decisions (see Figure 3).

### 3.6 Application status of multiregional cerebral oxygen monitoring

At present, multiregional cerebral oxygen monitoring has been applied in multiple clinical scenarios, such as anesthetic surgery, critical care, and neurological disease diagnosis and treatment. Based on the non-invasive and real-time characteristics of NIRS technology, multiregional cerebral oxygen saturation monitoring conducts continuous monitoring of oxygen saturation in multiple brain regions. Compared with conventional single frontal lobe monitoring, multiregional cerebral oxygen saturation monitoring can capture the spatial heterogeneity of cerebral oxygenation. Evaluating the oxygenation differences in multiple brain regions through NIRS monitoring is helpful for identifying brain tissue hypoxia areas caused by arterial occlusion, realizing synchronous monitoring of cerebral oxygen saturation between and within hemispheres, and revealing the oxygenation inhomogeneity between ischemic core areas and surrounding areas, thus guiding treatment decisions and evaluating the impact of treatment on oxygenation in multiple brain regions. For example, NIRS monitoring can identify local brain tissue hypoxia caused by arterial occlusion, helping doctors adjust treatment plans in a timely manner and avoid global misjudgment (Collette et al., 2022). At the same time, this capability solves the defect that single-region monitoring may miss local abnormalities and improves the sensitivity and specificity of monitoring. The latest relevant meta-analysis also reveals this problem: cardiopulmonary bypass cardiac surgery can induce cerebral microvascular dysfunction related to microthromboembolism events. If cerebral microembolism does not occur in the frontal cortex, NIRS may record false-negative results; that is, when severe ischemia occurs in other brain regions, the intraoperative rScO₂ value may still remain normal (Ding et al., 2023). At present, there have been applications of evaluating frontal and temporal cortical activation to detect anxiety and depression through NIRS technology (Lang et al., 2021). However, the clinical application of multi-channel and multiregional cerebral oxygen saturation monitoring is still in the initial stage. A study by Rummel et al. (2018) (2018, case series study, sample size *n* = 15, older adult patients undergoing cardiac surgery) found that spatially extended NIRS covering the vascular regions of the temporal lobe and parietal lobe can improve the detection sensitivity (40%) of severe cerebral hypoperfusion, and multi-channel continuous wave near-infrared spectroscopy has unique spatial and temporal resolution, which can be used to explore the local regulatory mechanisms of cerebral blood flow and metabolism. In critical care, multiregional cerebral oxygen saturation monitoring can prevent long-term neurocognitive sequelae. By monitoring multiple brain regions, such as the frontal and temporal sides, with NIRS, local oxygen desaturation can be identified and intervened in a timely manner to reduce the risk of cognitive impairment.

### 3.7 Core controversies in the application of NIRS-based multiregional cerebral oxygen monitoring in PND

See Table 3.

**Table 3:** Core controversies in the application of NIRS-based multiregional cerebral oxygen monitoring in PND of older adult surgical patients.

Core controversial issues	Supporting evidence	Contradictory evidence	Unresolved research gaps
1. Can multiregional NIRS monitoring improve the detection rate of PND in older adult surgical patients?	Multiregional monitoring can detect hypoxia in the non-prefrontal brain regions and improve monitoring sensitivity (Collette et al., 2022; Rummel et al., 2018); NIRS-guided management can reduce the incidence of early PND in non-cardiac surgery (Zhang C. Y. et al., 2024)	No significant difference in the overall incidence of PND in cardiac surgery; lack of evidence from large-sample randomized controlled trials (Semrau et al., 2021; Han et al., 2025)	Large-sample multicenter randomized controlled trials comparing multiregional and single-region NIRS in different surgical procedures; long-term follow-up of PND incidence (≥6 months)
2. Is there a unified surgical type/region-specific multiregional monitoring threshold?	Preliminary rScO₂ thresholds have been obtained for general surgery/thoracic surgery (a decrease of >20% from the baseline value, absolute value <50–60%) (Tsaousi et al., 2021; Cui et al., 2021)	Threshold differences exist in cardiac surgery/aortic dissection surgery; large individual differences in older adult patients (Han et al., 2025; Heuer et al., 2025)	Establish age/surgical type/region-specific thresholds through big data research; explore personalized monitoring thresholds based on cognitive reserve
3. Is there a direct causal relationship between multiregional NIRS monitoring and the reduction of PND?	Observational data support the association between the two; NIRS-guided management can reduce the incidence of early delirium (Holmgaard et al., 2019; Qiu et al., 2025)	Insufficient evidence from randomized controlled trials to confirm the causal relationship; hypoxemia is a risk marker rather than a direct cause (Semrau et al., 2021; Ding et al., 2023)	Mechanistic research on the relationship between multiregional oxygenation changes and PND; randomized controlled trials with hard endpoints (long-term cognitive function and mortality) as indicators
4. Is postoperative multiregional NIRS monitoring valuable for the intervention of PND?	Decreased cerebral oxygen saturation is common after cardiac surgery and is associated with long-term cognitive decline (Cioccari et al., 2021)	Few studies on postoperative multiregional monitoring; lack of intervention strategies for postoperative hypoxemia	Research on the duration of postoperative multiregional monitoring; intervention measures for persistent postoperative cerebral oxygen saturation decrease

### 3.8 Analysis of research heterogeneity in multiregional cerebral oxygen monitoring

The conclusions of existing studies on the application of multiregional cerebral oxygen monitoring in PND have significant heterogeneity. Observational studies mostly support that it can improve the detection sensitivity of cerebral hypoxia and is associated with the occurrence of PND, while randomized controlled trials have not confirmed that it can significantly reduce the incidence of PND. The root cause of this contradiction mainly includes methodological and pathophysiological factors, which are also key problems to be solved in future research.

#### 3.8.1 Methodological factors

(1) Surgical type heterogeneity: There are essential differences in the mechanisms of cerebral perfusion influence in different surgical types. Cardiac surgery, especially cardiopulmonary bypass surgery, has special factors, such as cerebral microthromboembolism and deep hypothermic circulatory arrest, and cerebral hypoxia is a mixture of global and local types, while cerebral hypoxia in non-cardiac surgery, such as orthopedics, abdominal surgery, and thoracic surgery, is mostly local hypoperfusion, leading to differences in the clinical value of multiregional cerebral oxygen monitoring in different surgeries. For example, its improvement effect on the overall PND in cardiac surgery is not significant, while its predictive value for postoperative delirium in orthopedic and thoracic surgery is higher.
(2) Non-uniformity of PND assessment: There are great differences in the assessment tools and time points of PND in existing studies. Some studies only assess delirium within 7 days after surgery, some assess delayed neurocognitive recovery within 30 days after surgery, and others focus on long-term cognitive decline 1 year after surgery. The impact of multiregional cerebral oxygen monitoring on PND at different time points may be different. Short-term delirium is related to acute abnormalities of cerebral oxygenation, while long-term cognitive decline is also related to factors such as neuroinflammation and neurodegenerative changes, leading to difficulty in integrating research conclusions.
(3) Differences in intervention thresholds and protocols: The intervention thresholds of cerebral oxygen saturation in studies are not uniform. Some studies adopt the “relative threshold (a decrease of >20% from the baseline)”, and some adopt the “absolute threshold (rScO₂ <50%)”. In addition, there are differences in the intervention protocols for cerebral oxygen desaturation in different studies, such as fluid replacement, vasopressors, and adjustment of anesthesia depth. The timeliness and effectiveness of intervention are different, which directly affect the PND-related outcomes, leading to the lack of comparability of research results.

#### 3.8.2 Pathophysiological factors

(1) Individual differences in cognitive reserve of older adult patients: There are significant individual differences in the cognitive reserve of older adult patients aged ≥65 years. Factors such as basic cognitive function, educational level, and underlying diseases (hypertension, diabetes, and cerebrovascular disease) will affect the tolerance of patients to abnormal cerebral oxygenation. Patients with higher cognitive reserve can tolerate transient cerebral oxygen desaturation, while patients with lower cognitive reserve are prone to PND. Existing studies have not conducted sufficient stratified analysis, leading to the masking of the effect of multiregional cerebral oxygen monitoring by individual differences.
(2) Heterogeneity of cerebral blood supply and metabolism in different brain regions: There are differences in the sources of vascular blood supply and metabolic demands in different brain regions of the brain. For example, the frontal lobe is supplied by the anterior cerebral artery and the middle cerebral artery, the temporal lobe by the middle cerebral artery, and the occipital lobe by the posterior cerebral artery. The risk of hypoxia in each brain region is different in different surgeries. However, the selection of monitoring brain regions in existing studies lacks standardization. Some studies only monitor the frontal lobe and the temporal lobe, and some monitor multiple regions of the whole brain, leading to differences in the correlation between monitoring results and PND.
(3) Synergistic effect of multiple perioperative factors: PND is a complication induced by multiple factors, and abnormal cerebral oxygenation is only one of the risk factors. Existing studies have not fully controlled other confounding factors, such as the selection of anesthetic drugs, postoperative analgesic protocols, and circadian rhythm disturbance. The intervention effect of multiregional cerebral oxygen monitoring is easily interfered with by other factors, making it difficult to reflect its independent impact on PND.

#### 3.8.3 Ideas for reducing research heterogeneity

Future research needs to reduce heterogeneity through a standardized research design, including: formulating standardized protocols for multiregional cerebral oxygen monitoring of different surgical types (clarifying monitoring brain regions and intervention thresholds); adopting unified PND assessment tools and time points, such as stratified assessment at 7 days, 30 days, and 6 months after surgery; conducting stratified research according to the cognitive reserve of older adult patients; strictly controlling other perioperative confounding factors; and carrying out multicenter, large-sample, placebo-controlled randomized controlled trials. At the same time, combined with pathophysiological mechanisms, the independent impact of multiregional cerebral oxygen monitoring on PND should be clarified.

### 3.9 Clinical translation of multiregional cerebral oxygen monitoring: monitoring algorithms and multimodal integration

The core of the translation of multiregional cerebral oxygen monitoring from basic research to clinical application is to establish a standardized monitoring algorithm and a multimodal neurophysiological monitoring integration system to realize the improvement of the precision, intelligence, and clinical practicability of cerebral oxygen monitoring.

#### 3.9.1 Standardized algorithm for multiregional cerebral oxygen monitoring

Combined with perioperative clinical needs, the standardized algorithm for multiregional cerebral oxygen monitoring should include: ① preoperative calibration algorithm: detecting the baseline rScO₂ value of each brain region before surgery, excluding extracranial interference factors such as anemia, abnormal CO₂, body position, and scalp fat thickness, to realize individualized calibration of the baseline value (Scheeren et al., 2012; Scheeren et al., 2012); ② abnormality identification algorithm: combining the relative threshold (a decrease of rScO₂ in each brain region by >20% compared with the baseline) and the absolute threshold (rScO₂ <50–60%) and identifying oxygenation abnormalities in a single brain region or multiple brain regions at the same time to distinguish focal and diffuse cerebral oxygen desaturation (Tsaousi et al., 2021; Cui et al., 2021); ③ stepwise intervention algorithm: initiating graded intervention according to the degree and scope of cerebral oxygen abnormalities, adopting measures such as fluid replacement and position adjustment for mild abnormalities, and timely vasopressor, optimizing anesthesia depth, and stopping surgical operations for severe abnormalities (Han et al., 2025; Heuer et al., 2025); and ④ postoperative follow-up algorithm: clarifying the optimal duration of postoperative multiregional cerebral oxygen monitoring and formulating postoperative brain protection plans according to the trend of cerebral oxygen recovery (Cioccari et al., 2021).

#### 3.9.2 Integration of multiregional cerebral oxygen monitoring and multimodal neurophysiological monitoring

Single NIRS monitoring is difficult to comprehensively reflect the brain function state. Integrating multiregional cerebral oxygen monitoring with technologies such as electroencephalography (EEG), transcranial Doppler ultrasound (TCD), and brain tissue oxygen partial pressure monitoring to construct a multimodal neurophysiological monitoring system can realize the synchronous assessment of cerebral oxygenation, electroencephalographic activity, and cerebral blood flow, significantly improving the comprehensiveness and accuracy of perioperative brain function monitoring (Bögli et al., 2025; Ryalino et al., 2024). The specific integration strategies include: ① multiregional rScO₂ combined with EEG: EEG can real-time reflect the abnormalities of electroencephalographic activity, and combined with cerebral oxygenation data, it can more accurately identify neuronal functional damage caused by cerebral ischemia and improve the sensitivity of early warning of PND (Ryalino et al., 2024); ② multiregional rScO₂ combined with TCD: TCD can monitor cerebral blood flow velocity, clarify whether the cause of abnormal cerebral oxygenation is cerebral hypoperfusion or abnormal oxygen consumption, and provide a basis for the formulation of intervention protocols (Hogue et al., 2021); and ③ multiregional rScO₂ combined with brain tissue oxygen partial pressure monitoring: brain tissue oxygen partial pressure is the gold standard for cerebral oxygenation, which can calibrate the NIRS monitoring results and improve its accuracy (Zhang C. Y. et al., 2024). The multimodal neurophysiological monitoring system has been initially applied in fields such as traumatic brain injury and cardiac surgery, and studies have shown that it can significantly reduce the incidence of postoperative neurological complications (Bögli et al., 2025). In the future, it is necessary to further optimize the application scheme of this system in older adult surgical patients to realize convenient clinical operation.

### 3.10 Cost–benefit and clinical feasibility analysis of multiregional cerebral oxygen monitoring

The clinical popularization of multiregional cerebral oxygen monitoring not only needs to verify its clinical value but also needs to evaluate its cost–benefit and clinical feasibility. From the cost perspective, the purchase cost of multi-channel NIRS monitoring equipment is higher than that of traditional single-channel equipment, but compared with the long-term medical expenses caused by postoperative PND, the decline in patients’ quality of life, and the social medical burden, it has significant long-term cost advantages (Khachaturian et al., 2020; Moore et al., 2022). Clinical studies have shown that reducing the incidence of postoperative delirium and PND through multiregional cerebral oxygen monitoring can shorten the hospital stay of patients, reduce the cost of postoperative rehabilitation treatment, and lower the long-term readmission rate (Roberts et al., 2021; Zhu et al., 2021), which has a good cost–benefit ratio from the perspective of health economics.

In terms of clinical feasibility, multiregional cerebral oxygen monitoring has the characteristics of non-invasiveness, simple operation, and no need for professional operation. The sensor probe can be quickly attached according to the brain anatomical region, and the real-time data can be integrated into the anesthesia monitoring system without increasing the workload of anesthesiologists during surgery (Kamar et al., 2024; Rummel et al., 2018). At the same time, with the development of technology, portable and high-precision multiregional NIRS equipment has been gradually developed, which further improves its application feasibility in different scenarios such as operating rooms, intensive care units, and postoperative wards (Cohen et al., 2025). In the future, it is necessary to further reduce the equipment cost, optimize the operation process, and promote its clinical application in primary hospitals.

### 3.11 Future development directions of multiregional cerebral oxygen monitoring

Although this new technology has equipment limitations such as poor sensitivity of monitoring and recording data and lack of unified monitoring thresholds, future research should continue to focus on the mechanism exploration and clinical application optimization in older adult surgical patients, correlate the occurrence of PND with the saturation changes in multiple brain regions, and further explore to clarify the key early warning indicators (Cohen et al., 2025).

At the same time, large-scale multicenter clinical trials should establish exclusive monitoring thresholds and standardized intervention processes for different surgical types, formulate intervention norms, establish surgical type-specific and region-specific multiregional monitoring thresholds, such as cardiac surgery vs. thoracic surgery and temporal lobe vs. parietal lobe, and standardized intervention protocols for older adult patients, and formulate evidence-based clinical intervention guidelines. The trials should also integrate the data of multiregional cerebral oxygen monitoring with other technologies such as electroencephalography (EEG) and transcranial Doppler into the multimodal neurophysiological monitoring system to construct a multimodal neurophysiological monitoring system, which enhances the precision of perioperative cerebral oxygen management and provides high-resolution assessment of cerebral perfusion and metabolism, which is helpful for early intervention of cerebral oxygen imbalance and reducing the risk of neurocognitive disorders (Bögli et al., 2025; Ryalino et al., 2024). In addition, combining multiregional cerebral oxygen monitoring with PND mechanism research and biomarker detection, such as IL-1β, ROS, and β-amyloid, can improve the accuracy of PND risk stratification in older adult surgical patients and help to clarify the causal relationship between multiregional oxygenation changes and the pathogenesis of PND; developing portable and high-precision multiregional NIRS monitoring equipment with strong anti-interference ability (resisting the interference of skin blood flow, temperature, and adipose tissue thickness) and optimizing the spatial resolution is the technical basis for its clinical popularization; finally, exploring the value of continuous postoperative multiregional cerebral oxygen monitoring in older adult surgical patients, especially intensive care unit patients, and establishing a whole-cycle monitoring system from intraoperative to postoperative is an important extension of its clinical application.

## 4 Summary

In summary, as an important extension of NIRS technology in the field of perioperative cerebral oxygen monitoring, the core value of multiregional cerebral oxygen saturation monitoring is to break through the spatial limitations of traditional single-region (frontal lobe) monitoring, realize real-time and non-invasive monitoring of oxygenation status in multiple cerebrovascular supply regions such as the temporal lobe and the parietal lobe through a multi-channel design, provide key technical support for accurate identification of cerebral hypoperfusion and early intervention of cerebral oxygen supply and demand imbalance, and has initially shown clinical application potential, providing a new management idea for perioperative neuroprotection in older adult patients. However, this technology still faces a series of challenges: the current relevant studies are mainly based on observational data and small-sample studies, and randomized controlled trials have not fully confirmed its direct causal relationship with postoperative cognitive dysfunction; problems such as the stability of equipment data quantification, lack of age/surgical type/region-specific multiregional monitoring thresholds for older adult patients, and insufficient clinical intervention guidelines limit its clinical popularization in older adult surgical patients.

Future research should focus on in-depth exploration of the causal relationship between multiregional oxygenation changes and the pathogenesis of PND, further optimizing the anti-interference ability and spatial resolution of the equipment, clarifying the monitoring standards and intervention processes for different surgical scenarios in older adult patients, establishing a whole-cycle multiregional monitoring system from intraoperative to postoperative, and promoting its transformation from the experimental exploration stage to a standardized routine clinical monitoring tool. Against the backdrop of the intensification of population aging, the maturity and application of multiregional cerebral oxygen saturation monitoring technology will provide important support for improving the perioperative safety of older adult patients, enhancing their postoperative quality of life, and reducing the social medical burden.

## Glossary

PND: Perioperative neurocognitive disorders
NIRS: Near-infrared spectroscopy
rScO₂: Regional cerebral oxygen saturation
POCD: Postoperative cognitive dysfunction
IL-1β: Interleukin-1β
ROS: Reactive oxygen species
BDNF: Brain-derived neurotrophic factor
TrkB: Tropomyosin-related kinase
HPC-mPFC: Hippocampal-medial prefrontal cortex
GABA: Gamma-aminobutyric acid
MMSE: Mini-Mental State Examination
MoCA: Montreal Cognitive Assessment
RBANS: Repeatable Battery for the Assessment of Neuropsychological Status
CAM: Confusion Assessment Method
DRS-R-98: Delirium Rating Scale-Revised-98
DCT: Delirium Cognitive Test
ERAS: Enhanced Recovery After Surgery
HbO_2_: Oxygenated hemoglobin
HHb: Deoxygenated hemoglobin
CBF: Cerebral blood flow
SjvO_2_: Jugular venous bulb oxygen saturation
PbtO_2_: Brain tissue oxygen partial pressure
EEG: Electroencephalography
PET: Positron emission tomography
fMRI: Functional magnetic resonance imaging
TCDm: Transcranial Doppler
CPR: Cardiopulmonary resuscitation
ROSC: Return of spontaneous circulation
OPCAB: Off-pump coronary artery bypass grafting
DHCA: Deep hypothermic circulatory arrest
CPB: Cardiopulmonary bypass
VATS: Video-assisted thoracoscopic surgery
OLV: One-lung ventilation
CEA: Carotid endarterectomy
TKA: Total knee arthroplasty
THA: Total hip arthroplasty
SSEPs: Somatosensory evoked potentials
